# Environmental DNA preserved in marine sediment for detecting jellyfish blooms after a tsunami

**DOI:** 10.1038/s41598-021-94286-2

**Published:** 2021-08-20

**Authors:** Mizuki Ogata, Reiji Masuda, Hiroya Harino, Masayuki K. Sakata, Makoto Hatakeyama, Katsuhide Yokoyama, Yoh Yamashita, Toshifumi Minamoto

**Affiliations:** 1 Maizuru Fisheries Research Station, Field Science Education and Research Center, Kyoto University, Nagahama, Maizuru, Kyoto 625-0086 Japan; 2 Benesse Corporation, 3-7-17 Minamigata, Kitaku, Okayama 700-8686 Japan; 3 Department of Human Sciences, Kobe College, 4-1 Okadayama, Nishinomiya, Hyogo 662-8508 Japan; 4 Graduate School of Human Development and Environment, Kobe University, 3-11 Tsurukabuto, Nada-ku, Kobe, Hyogo 657-8501 Japan; 5 Non-Profit Organization Mori-Umi, Nishi-Moune, Karakuwa, Kesennuma, Miyagi 988-0527 Japan; 6 Tokyo Metropolitan University, 1-1 Minami-Osawa, Hachioji, Tokyo 092-0397 Japan

**Keywords:** Environmental monitoring, Marine biology, Molecular ecology

## Abstract

Environmental DNA (eDNA) can be a powerful tool for detecting the distribution and abundance of target species. This study aimed to test the longevity of eDNA in marine sediment through a tank experiment and to use this information to reconstruct past faunal occurrence. In the tank experiment, juvenile jack mackerel (*Trachurus japonicus*) were kept in flow-through tanks with marine sediment for two weeks. Water and sediment samples from the tanks were collected after the removal of fish. In the field trial, sediment cores were collected in Moune Bay, northeast Japan, where unusual blooms of jellyfish (*Aurelia* sp.) occurred after a tsunami. The samples were analyzed by layers to detect the eDNA of jellyfish. The tank experiment revealed that after fish were removed, eDNA was not present in the water the next day, or subsequently, whereas eDNA was detectable in the sediment for 12 months. In the sediment core samples, jellyfish eDNA was detected at high concentrations above the layer with the highest content of polycyclic aromatic hydrocarbons, reflecting tsunami-induced oil spills. Thus, marine sediment eDNA preserves a record of target species for at least one year and can be used to reconstruct past faunal occurrence.

## Introduction

Environmental DNA (eDNA) refers to DNA fragments shed from organisms that remain in the environment and can be detected in several media such as water, sediment, soil, and air. The eDNA technology has been developed as an innovative approach, particularly for studying aquatic environments. Owing to the ease of field sampling, minimal disturbance to wild populations, and reliability in species identification, this technology is increasingly being used. Since Ficetola et al. detected American bullfrogs (*Rana catesbeiana*) in European ponds using eDNA^1^, the technology has progressed with easier DNA extraction and biomass estimation methods using quantitative PCR^2,3^, and eDNA metabarcoding using universal primers^4–7^.

To date, most eDNA studies have used water as the sample medium^1–11^. The sampling, extraction, and amplification of eDNA from water is a relatively simple procedure whereby a large volume can be processed by filtration. Because eDNA in water degrades within several days, it is useful for detecting organisms that were recently present. eDNA from freshwater fishes, such as the common carp *Cyprinus carpio* and bluegill sunfish *Lepomis macrochirus*, degrades at a rate of 10% per hour or 90% per day^8,9^. Thomsen et al. reported that among marine fishes, eDNA of *Platichthys flesus* degrades at 5% per hour and 32% per day, and would be undetectable after 6–7 days, whereas that of *Gasterosteus aculeatus* is undetectable within 0.9 days^10^. The detection window was found to be even narrower (less than 2 h) when tested in a marine environment using caged fish^11^. Therefore, eDNA in water can last from 1 h to a few weeks and only reflects the recent presence of target organisms unless it is transported from an adjacent large source. Although such a short longevity is useful to take a “snapshot” of a particular organism's distribution, it cannot be applied in a survey that aims to investigate the past several months.

Turner et al. reported that sediments in ponds and rivers contain a substantial amount of eDNA^12^. They also showed that eDNA of bighead Asian carp *Hypophthalmichthys* spp. can be detected in sediment up to 132 days after the removal of these fish. Their study suggested that eDNA in sediments can be used to detect the presence of past organisms. In studies of ancient DNA, such techniques have been applied to reconstruct several thousand-year-old flora from lake sediment^13,14^. Because eDNA can be preserved in sediment for long periods, this technology is expected to be applicable to studies that use samples that are more than a few weeks old. Historical faunal data can be valuable for studying future faunal occurrence and for planning ecosystem management. Studies on sediment eDNA are flourishing in freshwater systems^12,15–17^, deep seas^18–20^, and shallow marine environments^21–25^. Most marine sediment eDNA studies use metabarcoding to detect species or reconstruct community structures. However, there are only two publications that have successfully reconstructed the abundance of specific macro-organisms from marine sediment eDNA^21,22^. Furthermore, the longevity and decay of sediment eDNA under controlled conditions have only been tested in freshwater ponds^12^, sample sediment collected from a biotope^26^, or a combination of quartz sand and artificial seawater in beakers^27^. However, to the best of our knowledge, no study has attempted to monitor the accumulation and reduction process of eDNA in natural marine sediment with flow-through seawater.

**Figure 1:**
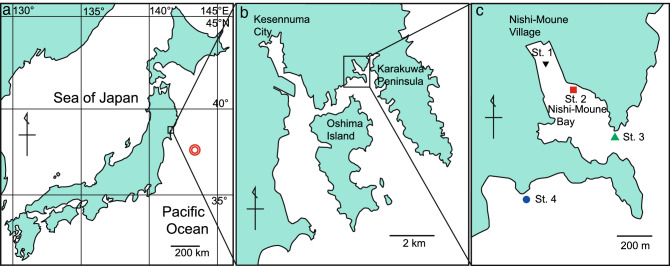
Sampling locations of sediment cores in northeast Japan. Double circle in (a) represents the earthquake epicenter. Adobe Illustrator CC (ver. 22.1, https://www.adobe.com/jp/products/illustrator.html) was used to create the maps.

**Figure 2:**
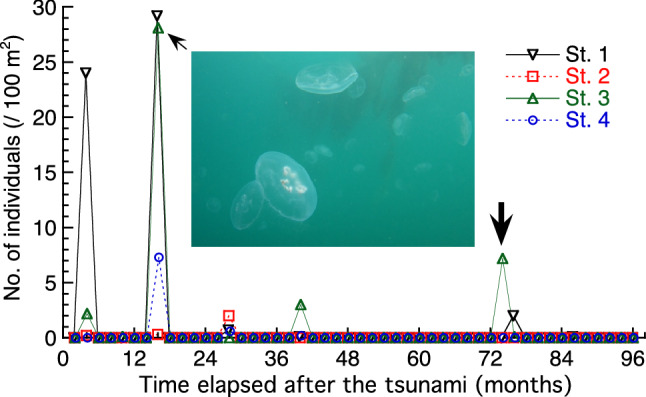
Mean number of moon jellyfish (*Aurelia* sp.) in ten transects (2 m × 50 m each) detected at the four stations in and around Moune Bay, Kesennuma, in the visual surveys over the eight years after the 2011 tsunami (Masuda et al.^29^ with updated data). Station numbers correspond with those in Fig. 1. Inserted picture was taken on July 2012 at St. 3. The thick arrow represents the timing of sediment core collection in the present study.

In March 2011, the coastal area of northeast Japan suffered from a large earthquake followed by a massive tsunami (the 2011 Great East Japan Earthquake and Tsunami, Fig. 1). The tsunami induced large-scale oil spill related fires in some areas, such as Kesennuma^28^, devastating the coastal fauna. Underwater visual surveys that started two months after the tsunami revealed that the recovery of fauna was initiated by the outbreaks of short-lived animals such as jellyfish, after which numerical expansion of short-lived gobies occurred, followed by gradual increases in the number of longer-lived animals such as rockfish, sea cucumbers, and abalones^29^. Notably, blooms of moon jellyfish *Aurelia* sp. occurred during the first two years after the tsunami (Fig. 2), and sea nettle *Chrysaora pacifica* was also sporadically recorded during the same period (Supplementary Table S1). Nevertheless, such a post-event survey merely describes the phenomena that followed the event, whereas the status of fauna before the event is speculative and/or anecdotal. Sediment eDNA has the potential to provide information about fauna prior to such an ecological event. Chemical signatures of the oil spill in sediment cores can be utilized to trace the timing of the tsunami.

The principal objectives of this work were to evaluate how eDNA is accumulated and preserved in marine sediment, and to attempt the retrieval of eDNA from sediment core samples. The tank experiment showed that eDNA of jack mackerel *Trachurus japonicus* is preserved for over 12 months. Core samples from the sea bottom were then collected six years after the tsunami and eDNA was extracted from sediment layers and analyzed to determine whether pre- and post-tsunami fauna were identifiable. We originally aimed to identify fishes, however the fish eDNA retrieved from the sediments was in low concentration. Therefore, we decided to target jellyfish, as eDNA emissions per biomass in jellyfish is approximately 700 times greater than that in fish^30^. It has also been confirmed that eDNA detected in seawater correlates with visually estimated biomass in both fish and jellyfish^30^; thus, the results of the tank experiment using fish would be applicable to the field study using jellyfish eDNA. As a signature of the tsunami, polycyclic aromatic hydrocarbons (PAHs) were quantified because they are the most commonly used criteria for measuring the environmental impact after oil spills^31^. The scale of oil spills, as a consequence of the tsunami in Kesennuma, was estimated to be 7530 kL of marine diesel^28^, which was exceptionally high for this area. Conspicuous post-tsunami faunal records, i.e., jellyfish blooms, can thus be detected from the sediment eDNA.

## Results

### Detection of eDNA in tank water and sediment after fish introduction

**Figure 3:**
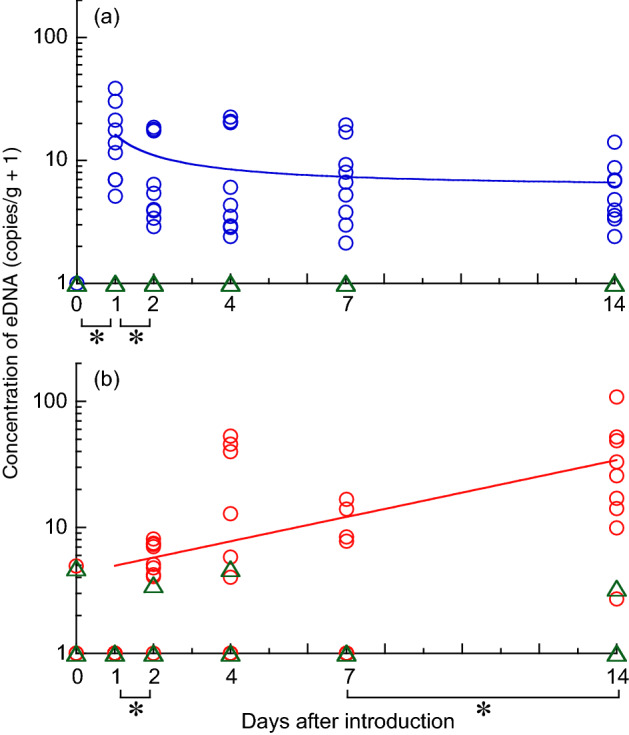
Concentration of eDNA in (**a**) tank water and (**b**) sediment after the introduction of fish. Nine samples (triplicates from each of three tanks, indicated by circles) for test groups and three samples (triplicates from one tank, triangles) for control were analyzed and plotted. Asterisks indicate significant differences between two consecutive sampling days (*p* < 0.05, Tukey’s HSD test). The best models shown in Supplementary Table S3 were fitted.

The concentrations of eDNA in all nine samples of water collected prior to fish introduction were undetectable, whereas all samples were positive when jack mackerel was present in the tanks (Fig. 3a, Supplementary Table S2). In the sediment samples, the number of positive samples gradually increased after the introduction of fish, and all samples were positive 14 d after fish introduction (Fig. 3b). The mean ± SD eDNA concentration in the water on day 14 after fish introduction was 5.1 ± 3.6 copies/g (range: 1.4–13.1), whereas that in the sediment was 33.6 ± 32.3 copies/g (1.7–107.2).

The concentration of eDNA detected in the water was significantly different among days after fish introduction (*p* < 0.001, ANOVA). Comparisons between each combination of consecutive sampling days revealed a significant increase from day 0 (prior to the introduction) to day 1 (*p* < 0.001) and a significant decrease from day 1 to day 2 (*p* = 0.02, Tukey’s HSD test; Fig. 3a). No significant differences were detected after day 2. The reciprocal model best explained the stabilization of eDNA concentration (Supplementary Table S3).

The concentration of eDNA in the sediment was also significantly different among days (*p* < 0.001, ANOVA). A significant increase was detected from day 1 to day 2 (*p* = 0.02) and from days 7 to 14 (*p* < 0.001, Tukey’s HSD test; Fig. 3b). The concentration of eDNA in the sediment after the introduction of fish best fit the exponential model, representing a gradual increase (Supplementary Table S3).

### Decrease in eDNA concentration in tank water and sediment after fish removal

**Figure 4:**
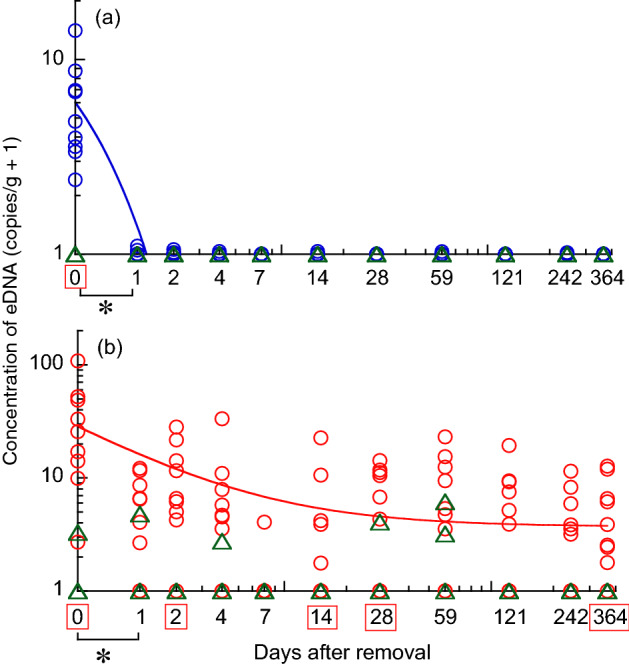
Concentration of eDNA in (**a**) tank water and (**b**) sediment after the removal of fish. Circles and triangles represent test and control tanks, respectively. Asterisks indicate significant differences between two consecutive sampling days, and squares indicate significant differences between test and control tanks on the same sampling day (*p* < 0.05, ANOVA). The best models in Supplementary Table S3 were fitted.

The concentration of eDNA in tank water declined immediately after the removal of fish, and positive values were only detected in 0–2 of 9 samples on any sampling day (Fig. 4a). The concentration in the test tanks was only significantly higher than that in the control tank on day 0 after the fish were removed (*p* < 0.001, ANOVA), whereas no significant difference was detected between the test and control tanks on any subsequent day. The log Y equation best fit this process (Supplementary Table S3).

In contrast, although the eDNA concentration in the sediment declined after the removal of fish, it was still detected in 5–8 of 9 samples on all days except on day 7, when only one sample was positive. Eight out of nine samples were positive for eDNA 12 months after fish removal; mean ± SD concentrations of eDNA in these samples were 4.4 ± 4.3 copies/g (range: 0–11.7). The sediment eDNA concentrations in the test tanks were consistently higher than that in the control. The difference between test and control tanks was significant on days 0 (*p* < 0.001), 2 (*p* < 0.01) and 14 (*p* < 0.01), and in month 1 (*p* < 0.001) and 12 (*p* = 0.02, ANOVA; Fig. 4b). The difference was marginal on days 1 (*p* = 0.055) and 4 (*p* = 0.096), and in month 8 (*p* = 0.107). The reciprocal model best explained the reduction in sediment eDNA, representing a rapid decrease in eDNA concentration after the removal of fish, followed by relatively consistent values that converge to an asymptote (Supplementary Table S3).

Concentrations of eDNA in the control tank water were consistently below the detection level across all samples in the post-introduction and post-removal periods (*n* = 18 and 33, respectively). Sediment in the control tank sporadically contained eDNA with mean ± SD concentrations of 0.68 ± 1.35 copies/g (range: 0–3.78) and 0.55 ± 1.29 copies/g (range: 0–5.14) in the post-introduction and post-removal periods, respectively (Supplementary Table S2).

### Analysis of sediment core samples by layers

Concentrations of eDNA were analyzed for all three core samples collected at each station (12 cores in total, named Core St. 1–1…St. 3–3). PAHs were analyzed for one core at St. 1 and all three cores at St. 3, where peaks of jellyfish eDNA were clear and the lengths of cores were long enough.

**Figure 5:**
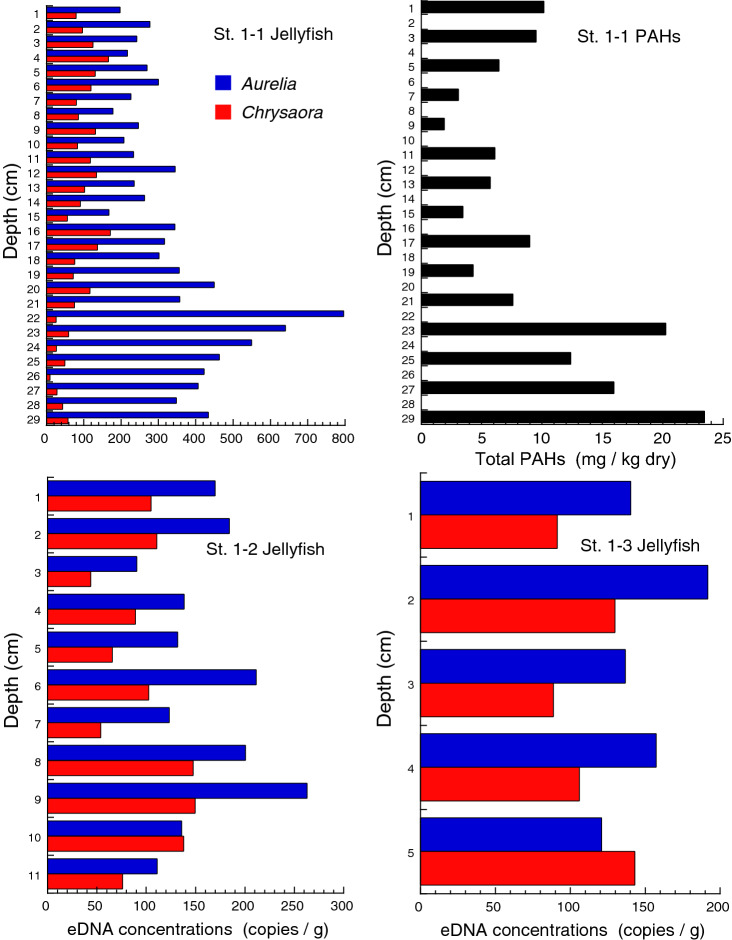
Concentrations of moon jellyfish (*Aurelia* sp.) and sea nettle (*Chrysaora pacifica*) eDNA and polycyclic aromatic hydrocarbons (PAHs) analyzed from samples of the sediment cores collected at St. 1 in Moune, Kesennuma, northeast Japan. Three cores were analyzed for jellyfish eDNA, whereas PAHs were analyzed only for the longest one (Core St. 1–1).

**Figure 6:**
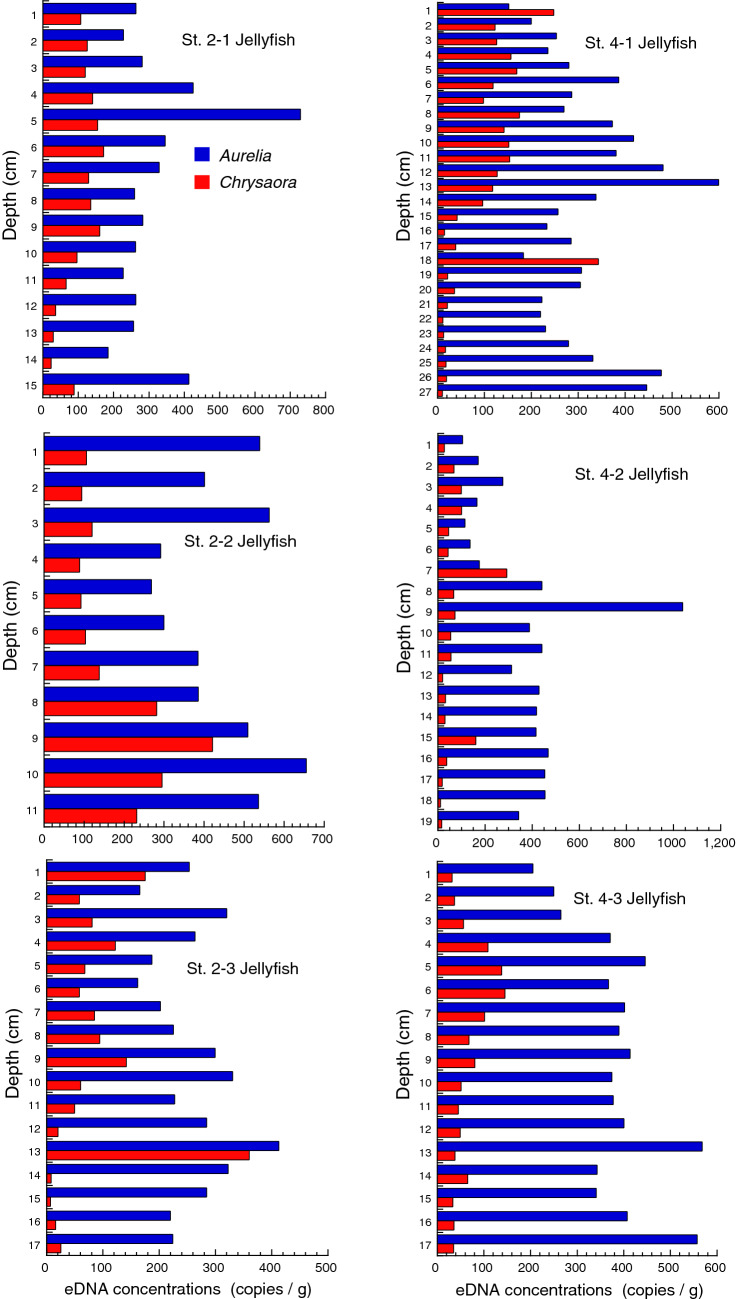
Concentrations of moon jellyfish (*Aurelia* sp.) and sea nettle (*Chrysaora pacifica*) eDNA analyzed from samples of the sediment cores collected at St. 2 and St. 4 in Moune, Kesennuma, northeast Japan. Only eDNA was analyzed for these cores.

**Figure 7:**
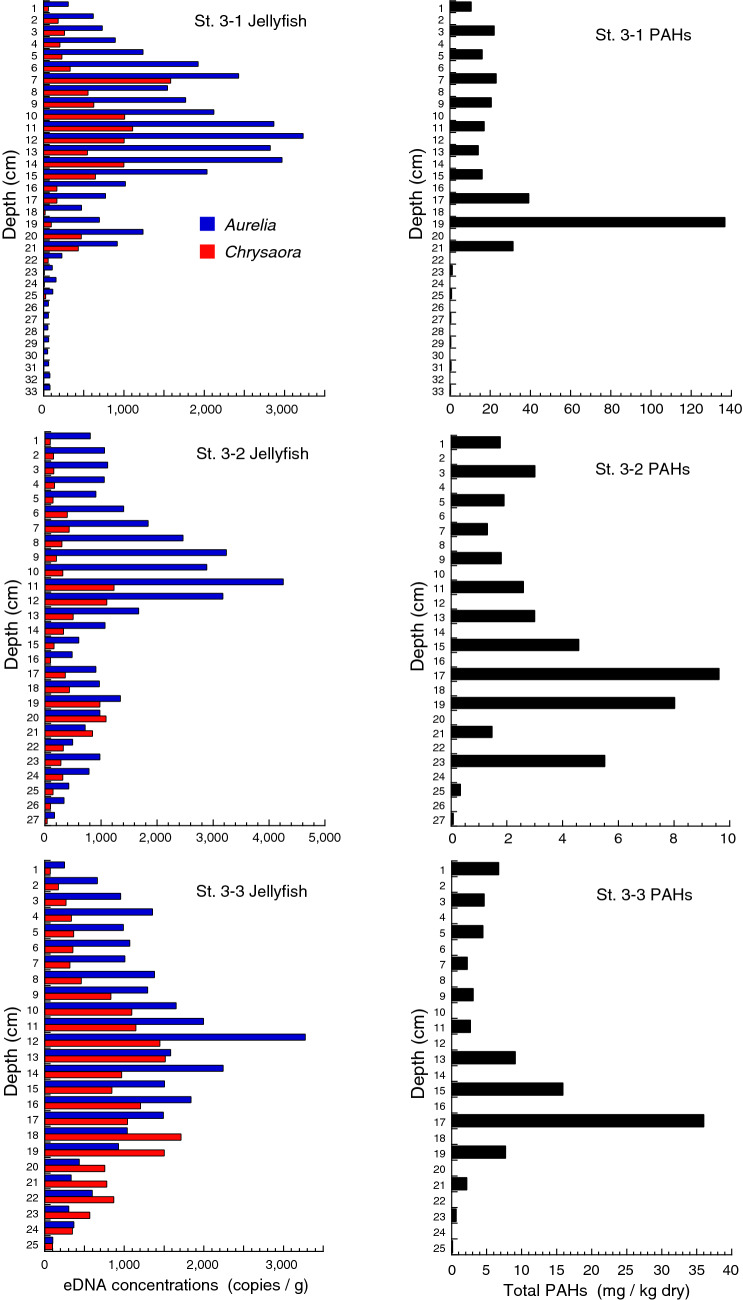
Concentrations of moon jellyfish (*Aurelia* sp.) and sea nettle (*Chrysaora pacifica*) eDNA and polycyclic aromatic hydrocarbons (PAHs) analyzed from samples of the sediment cores collected at St. 3 in Moune, Kesennuma, northeast Japan. Both eDNA and PAHs were analyzed for all three cores.

eDNA of both jellyfish species was detected in every layer of the sediment core samples, but the amount varied among layers (Figs. 5, 6, 7, Supplementary Table S4). In the core samples collected at St. 1, located in the inner bay, eDNA concentrations of moon jellyfish were generally high in the lower layers, with the highest concentrations detected in the 22 cm depth layer of the longest core (core 1–1, Fig. 5). eDNA concentrations of sea nettle were higher in the layers that were at a depth of 21 cm or shallower, and peaked at a depth of 16 cm in the longest core. The highest concentration of PAHs in core 1–1 was detected at 29 cm, the deepest layer of the core, and the second highest PAHs concentration was at 23 cm. Therefore, most of the core samples collected here are likely to have undergone post-tsunami sedimentation. Jellyfish eDNA concentrations did not show any clear peaks in the cores collected at St. 2 and 4 (Fig. 6). The peak concentrations in cores collected at St. 3 were generally higher than those at other stations (Fig. 7). Moon jellyfish eDNA showed peak concentrations at depths of 12, 11, and 12 cm for cores 3–1, 3–2, and 3–3, respectively, whereas those of sea nettle were not as clear, with the highest concentrations at depths of 7, 11, and 18 cm for the same cores. The peak PAH levels in the cores at St. 3 were at depths of 19, 17, and 17 cm, for cores 3–1, 3–2, and 3–3, respectively.

**Figure 8:**
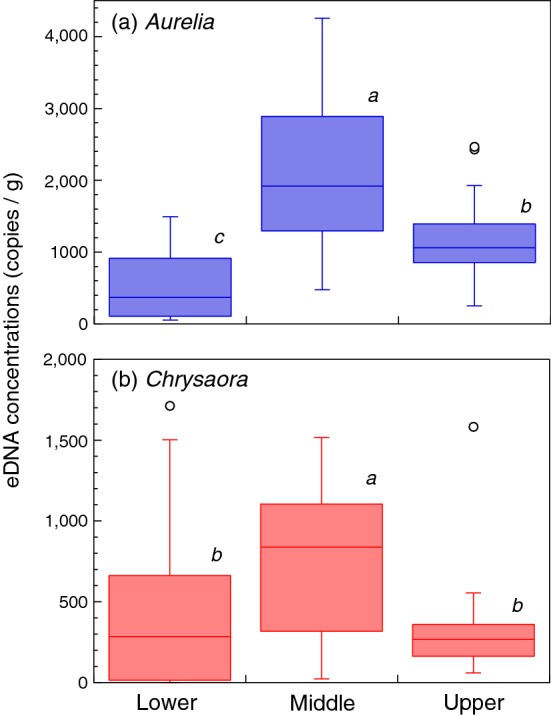
Concentrations of eDNA were compared among the lower (the peak of the polycyclic aromatic hydrocarbons (PAHs) and below), middle (lower half of the layers above the peak of the PAHs), and upper parts of the core layers (the upper rest) collected at St. 3. Different letters indicate significant differences among parts (*p* < 0.05, Tukey’s HSD test).

When the cores at St. 3 were divided into three parts, the middle part was found to contain a higher concentration of moon jellyfish eDNA than the upper and lower parts (*p* < 0.001, Tukey’s HSD test), with the upper part containing more eDNA than the lower part (*p* = 0.002, Fig. 8a). For sea nettle, the middle part contained a higher eDNA concentrations than both the upper (*p* < 0.001) and lower parts (*p* = 0.001), whereas there was no difference in eDNA concentrations between the upper and lower parts (*p* = 0.44, Fig. 8b).

## Discussions

We found that eDNA in tank sediment gradually increased in our seawater experimental system, and it was detectable up to 1 year after the removal of fish. The analysis of sediment core samples enabled us to detect jellyfish blooms after the tsunami, which occurred 6 years prior to the sampling.

### Dynamics of eDNA in the tank experiment

A high concentration of eDNA was detected in tank water on day 1 after fish introduction, after which its concentration declined and stabilized. A similar phenomenon was observed in other studies that reported high eDNA concentrations on the first 1–2 days after introduction of common carp^2^ and bluegill sunfish^9^, and these results were attributed to handling stress when fish are first introduced into tanks. After the removal of fish, eDNA decreased drastically because the water was consistently exchanged. These results imply that eDNA extracted from field-collected water is likely to represent either the most recent presence of the target species in a survey area, or a relatively recent event of the target species, such as predation and spawning, in a nearby area.

Sediment eDNA in the tank gradually increased after the introduction of fish. Some of the released eDNA could have been deposited on the tank bottom and may have accumulated to build up a detectable amount on day 2 or later after the introduction of fish. This implies that sediment eDNA collected in the field would reflect more resident organisms, rather than transient ones. Our results are in accordance with those of Wei et al.^21^, who suggested that amphipod eDNA in sediment represents the average state of abundance of amphipods in the environment. Although eDNA concentration in the sediment declined from day 0 to day 1 after the removal of fish, it was detectable for the following 12 months. The rapid decrease in sediment eDNA from day 0 to day 1 was in accordance with that in the water. This reduction process may be due to the removal of eDNA by running water, other physical processes, or biochemical decomposition. After this initial reduction, sediment eDNA could be preserved for a considerable amount of time. Turner et al. reported that eDNA in sediment was detectable in a freshwater pond 132 days after the fish were removed^12^. Persistence of eDNA has also been reported for 28 days in biotope sediment^26^ or 20 days in 250 mL beakers^27^. Our study confirmed the persistence for a longer period (12 months) under a flow-through seawater system, with a wide range of temperature fluctuations that are likely to facilitate the decomposition of eDNA. In field studies, Kuwae et al. successfully quantified the eDNA of three fish species in marine sediments spanning 300 years^22^. Ancient DNA can be preserved for tens of thousands of years in sediment under suitable conditions^32^. Overall, the preservation of eDNA in sediment tends to be much better than that in water.

The rapid increase in eDNA concentration from day 1 to day 2 after the introduction of fish as well as the lower detected eDNA concentration on day 7 after the removal of fish may have partly been due to an uneven distribution of eDNA in the sediment. Such a patchy distribution could have been induced by the water current in the experimental tank. Fish feces generally sink and can thus be a source of large amounts of eDNA present in a patchy distribution. During the collection of sediment samples from the tanks, we paid attention to counterbalance the eDNA distribution and tried to minimize the effect of variations in its distribution. The patchy distribution of sediment eDNA, as opposed to a relatively homogenous distribution of water eDNA, was confirmed in samples collected from a natural river^16^.

eDNA in the water of the control tank was always below the limit of detection, whereas sediment eDNA in the control tank had a positive value at several instances. The source of eDNA in the control tank sediment was most likely the supplied seawater. Jack mackerel is abundant along the pier where the seawater was pumped from, and it is known that eDNA is occasionally detected in seawater even after being passed through the fine sand filtration system^33^. Therefore, although we did not detect eDNA when we sampled the supplied water in the present study, occasional intrusion of eDNA may have resulted in its accumulation to a detectable level. Furthermore, while we could not exclude the possibility that jack mackerel eDNA was initially present in the collected sediment, its concentration should have been very low as jack mackerel usually live around reefs, whereas the sediment was collected from the muddy bottom without a reef. In the present study, the reduction of eDNA in the sediment was confirmed by a comparison with the control tank. The concentration in the control tank was extremely low [0.60 copies/g in the control vs 8.66 copies/g in the test tanks (Supplementary Table S2)], and therefore regression curves were drawn without considering this.

### Identifying jellyfish blooms by eDNA in sediment cores

In the longest sediment core collected at St. 1, the peak layer containing moon jellyfish eDNA was identified slightly above the peak layer of PAHs, which was recorded in the deepest layer of the core. The sampling location was relatively shallow (8.3 m in depth) and a river consistently flowing into the bay transported the sediment, including pebbles. The content of eDNA in sediment with a large particle size tends to be low primarily because of its relatively small surface area^32^. In the present study, physically unstable environmental conditions may have removed the signature of the tsunami that occurred six years prior to sediment sampling. Furthermore, benthic animals such as sea cucumbers, mud shrimps, and polychaetes were abundant in this area^29^ and may have contributed to the destruction of layers and decomposition of eDNA.

At St. 3, the peak layer of moon jellyfish eDNA was recorded just above the peak layer of PAHs. Layers below a depth of 17–19 cm should correspond to pre-tsunami sediment. The location of sampling was relatively deep (depth of 23 m) and may have been under stable conditions, thus preserving eDNA records of jellyfish blooms. Concentrations of moon jellyfish eDNA were higher in the middle part of the core than in the upper part, coinciding with the visual surveys (Fig. 2). Interestingly, the eDNA concentration was higher in the upper part than in the lower part of the core (Fig. 8a). This implies that moon jellyfish were more abundant in recent years than before the tsunami occurred. Indeed, their blooms, although less dense than those immediately after the tsunami, could be visually detected six years after the tsunami, but were no longer detected 7–10 years afterwards (Fig. 2; Supplementary Table S1). It is possible that older eDNA in deeper sediment layers had been degraded. However, DNA decomposition is mainly attributable to microbial activity which is typically reduced in low oxygen conditions such as in deep sediment^34–36^. Our tank experiment also revealed consistent eDNA detection in sediment from days 1 to 364 after the fish were removed. Therefore, it is reasonable to assume that relatively low eDNA concentrations in deeper core samples represent a lower abundance of jellyfish. However, we cannot extrapolate our tank experiment to the interpretation of sediment core samples because the time scale of the former was shorter than that of the latter. Quantification of total eDNA and/or eDNA targets of offshore pelagic species that are expected to be relatively consistent before and after the tsunami may support the validity of the above argument.

The impact of the tsunami was expected to be stronger in the inner bay (St. 1) compared to other locations, and we therefore expected to detect a clear signature of the tsunami at St. 1; however, we failed to detect such a signature. The average depth of sampling at St. 3 (23.0 m) was deeper than that at other stations (8.1, 9.6, and 14.0 m at stations 1, 2, and 4, respectively). Considering relatively high physical and biochemical disturbances in shallow waters, future sediment core samples should be taken from deeper areas.

Sea nettle eDNA was variable and its peak layers were difficult to detect. In our visual surveys, although individual sea nettles were observed near the sea surface two months after the tsunami, underwater detection was sporadic (Supplementary Table S1). The lack of a clear bloom of this species may explain the unclear eDNA peak. Nevertheless, concentrations of sea nettle eDNA in the middle part of the sediment core were higher than those in the upper or lower parts, and those in the latter two parts were not significantly different from each other (Fig. 8b). This suggests that at the time of sediment core sampling, the sea nettle density was at pre-tsunami levels.

Considering the relatively low number of individuals, the amount of sea nettle eDNA detected in the present study was substantial. Previous studies showed that although the detected amount of sea nettle eDNA clearly reflected the seasonal appearance of this species as visually documented along a pier^37^, their eDNA also tended to be abundant when this species was not detected in underwater visual censuses^30^. Sea nettles with large body sizes (approximately 30 cm in diameter and 3 m in tentacle length) can be a major source of eDNA, even from a single individual. Alternatively, the seasonal appearance of small but abundant ephyrae can be missed by underwater visual censuses, but their eDNA is likely to be retained in the sediment. Underestimation of gelatinous plankton in conventional survey methods compared to the eDNA approach has been demonstrated in a study that applied eDNA metabarcoding to mesopelagic zone water samples^20^.

Oil spills caused by the tsunami should have caused a temporary deterioration in local environmental conditions as a consequence of the spread of toxic chemicals and induction of hypoxia. Indeed, dead fish and abalones were found in underwater visual surveys conducted two months after the tsunami^38^. *Aurelia* jellyfish have short and flexible life cycles^39^, are opportunistic, and can survive at low oxygen levels^40^. Therefore, they are at an advantage over other organisms in hypoxic, and other usually unfavorable environmental conditions. The present study provided evidence that jellyfish increased their population size immediately after the tsunami, when the environment was not suitable for other marine animals. Jellyfish blooms combined with high input of nutrients can modify planktonic assemblages and increase red tide-forming dinoflagellates^41^. However, in our survey area, jellyfish blooms were temporary, while other macro-invertebrates and fishes gradually increased their abundance in subsequent years^29^. Oyster cultures resumed within a year after the tsunami^42^ may have also contributed to the recovery of the ecosystem; bivalves are efficient plankton feeders that remove excessive nutritional load, resulting in less nutritional input for jellyfish.

## Conclusions and perspectives

The present study demonstrated the potential use of sediment eDNA for obtaining past faunal information. Ideally, the impact of natural and anthropogenic disturbances on ecosystems should be evaluated by pre- and post-surveys in both impacted and control sites. However, such a study design requires consistent monitoring in any context of interest, which can have financial and logistical constraints. Alternatively, sediment eDNA may be used to survey biological information from the past. The present study focused on two species of jellyfish, but the targets species can theoretically be expanded to include any animals and plants depending on the amounts of recovered eDNA and available primer and probe sets.

In freshwater systems fish eDNA obtained from the water usually yields better results than sediment samples^15,17^. Similarly, in marine environments, fishes and gelatinous plankton are represented better in water than in sediment eDNA, and the opposite is true for benthos^19,23,24^. In our pilot study, we also tried to detect fishes by analyzing sediment cores for eDNA using species-specific primers, but the results were ambiguous partly due to the limited amount of extracted DNA. Unlike eDNA in water where large amounts can be filtered, eDNA in sediment has a limited sample amount from which DNA can be extracted. Nevertheless, Sakata et al. reported an equivalent detection of fish species from river water and sediment samples using universal fish primers^26^. Universal primers should be used in future studies to detect fish eDNA in sediment cores. Additionally, migratory species with seasonally high abundances that were missed by either visual censuses or water eDNA extraction, could be detected in sediments by using eDNA metabarcoding. Meanwhile, further innovation is required to enable the application of sediment eDNA to a wider range of ecological contexts.

## Materials and methods

### Ethics statement

Field research, including sediment collection, was approved by the Harbormaster of Maizuru Bay (Permission Number 31 issued on July 1, 2016). All the experiments were performed in accordance with the guidelines on the Regulation on Animal Experimentation of Kyoto University (https://www.kyoto-u.ac.jp/en/research/research-compliance-ethics/animal-experiments), the Kyoto Prefecture Fishery Management Rules (http://www.pref.kyoto.jp/reiki/reiki_honbun/a300RG00000634.html), and the ARRIVE guidelines (https://arriveguidelines.org). Fish (15 individuals of jack mackerel juveniles) were anesthetized prior to length and weight measurements using 0.05% of 2-phenoxyethanol, and all individuals recovered from the anesthesia. No fish were sacrificed or injured in the present study. The research plan was approved by the institutional review boards at the Maizuru Fisheries Research Station (MFRS) of Kyoto University.

### Detection of eDNA in the water and sediment in experimental tanks

On July 6, 2016, natural marine sediment was collected at a depth of 47 m off the shore of Kyoto in the Sea of Japan (35.5544° N, 135.3210° E), using a Smith–McIntyre bottom sampler. Approximately 100 L of sediment was collected from 13 casts and was preserved in four large, covered containers at room temperature until the experiment began. Four sub-samples, 3 g from each container, were used for eDNA extraction and detection of jack mackerel using the method described below, and we confirmed that none of them contained the DNA of this species. The median particle diameter of the sediment was 47.7 μm, and mud content was 61.9% based on analyses using a laser diffraction particle size analyzer (SALD-2200, Shimadzu, Kyoto, Japan). Jack mackerel juveniles were collected by hook-and-line fishing from the pier of the MFRS. This species is the most abundant fish in this area and is typically found in waters 14 °C or warmer^43^.

Four 200 L polycarbonate tanks (66 cm in bottom diameter) were set in the rearing facility of MFRS; three were used as test tanks, and the fourth was used as a control (blank) tank. The marine sediment (24 L, 7 cm in thick layer) was placed in each tank. Fine-filtered seawater was provided 2 d after the sediment had settled. The seawater used was pumped from 6 m depth offshore from the MFRS and filtered by passing through coarse polyvinyl fabric and fine sand of ca. 0.6 mm in diameter (5G-ST, Nikkiso Eiko, Japan; www.nikkiso-eiko.co.jp). Water was supplied at the rate of 490 mL min^−1^ (four cycles of circulation per day) and was drained from the center of each tank, filtered through a 2 mm mesh net. Aeration was performed at a rate of 600 mL min^−1^. This flow-through system was maintained throughout the experimental period.

Five individual jack mackerel (70.7 ± 4.4 mm in total length and 3.26 ± 0.67 g in wet mass, mean ± SD) were introduced in each of the three test tanks on August 8, 7 days after the sediment was introduced. Defrosted krill *Euphausia pacifica* (5 g per tank) was fed to the fish between 16:00 and 17:00 every day. Fish were removed from the tanks 14 days after introduction using two hand nets, taking care not to disturb the sediment. Water temperature was recorded using a digital thermometer at 10:00 every day while the fish were kept in the tanks, for the following 14 days after their removal, and once a week thereafter. The water temperature ranged from 24.9 to 29.5 °C (mean = 27.9 °C) during the first four weeks of the experiment and from 9.4 to 27.9 °C (mean = 17.8 °C) during the following months. These conditions are similar to the natural condition that would be undergone by eDNA in sediment; for the last 19 years, the recorded bottom water temperature in the area from which the jack mackerel had been collected ranged from 8.5 to 29.6 °C, with a mean of 18.2 °C (Masuda 2008^43^ with updated data). All rearing equipment was either newly purchased or bleached with 0.1% sodium hypochlorite and rinsed well with tap water before use.

Water and sediment samples were collected immediately before the introduction of fish (day 0) and on days 1, 2, 4, 7, and 14 after their introduction. Sampling was also conducted on days 0, 1, 2, 4, 7, and 14, as well as in months 1, 2, 4, 8, and 12 after the removal of fish. Three 1 L water samples and three sediment samples (3 g) were collected from each tank on each sampling day. Water was collected from the drainage outlet in plastic bottles, and sediment was collected in petri dishes (inner diameter of 58 mm and depth of 21 mm). The sediment was collected by pushing the open end of a petri dish onto the surface of the sediment and securing the cover from underneath. Sediment sampling was conducted with a pair of prebleached long-sleeved gloves. One sample was obtained from the central tank area, and another two from near the peripheral tank area. Repetitive collection from the same location was avoided by marking each sampling location with a piece of PVC pipe (similar in diameter to the petri dishes and 3 cm in height).

Water was filtered using glass fiber filters (0.7 μm mesh; GF/F 47 mm, GE Healthcare Japan, Tokyo, Japan). This mesh size, along with 0.45 μm, are two of the most commonly used filters in macroorganism eDNA studies^44^. The amount of eDNA detected using a 0.7 μm mesh is equivalent to that by a 0.45 μm mesh^30^. Contamination was evaluated by filtering 1 L of reverse osmosis water at the end of each sampling day. The filtered paper was wrapped in aluminum foil and preserved at − 20 °C.

### Sediment core sampling

Sediment core samples were collected at four locations (St. 1–4) in and around Nishi-Moune Bay, Kesennuma, Miyagi, Japan (38.8919–38.8932°N, 141.6235–141.6262°E; Fig. 1) on May 20, 2017. St. 1 was in the inner part of the bay where the tsunami impact was assumed to be the highest, with a run-up height of 15 m. St. 2 was located along a shallow rocky shore where the tsunami impact was limited. St. 3 was located at the mouth of the bay, and St. 4 was outside the bay. Average depths of the seafloor where cores were collected were 8.1, 9.6, 23.0, and 14.0 m at stations 1, 2, 3, and 4, respectively. Seafloor temperatures ranged from 9.9 to 11.5 °C. An acrylic pipe (inner diameter of 54 mm, length of 50 cm, and thickness of 3 mm) was pushed into the bottom sediment by a scuba diver. A silicon cap (59 and 52 mm in upper and lower diameter, respectively, and 45 mm in height) was placed on the top of the pipe, and the diver slowly pulled the pipe up and put another cap on the bottom. Three cores were collected from each location and transferred to a boat at the sea surface. Sediment core samples were kept vertical to avoid disturbing the layers and protected from direct sunlight. Cores were immediately transferred to the laboratory within 10 min, and were prepared for the cutting process.

The core samples (1 cm thickness) were cut by layers as follows: after removing the bottom cap, a core sample pipe was placed on a stage that pushed the sediment inside. Seawater in the upper part of the pipe was discarded until the top of the sediment appeared on the surface. A thin acrylic plate was used to cut the core, and the cut specimen was placed in a small vinyl bag and preserved at -20 °C. All 12 collected cores were used for eDNA analysis, and one at St. 1 (inner bay) and all three at St. 3 (bay mouth) were used for the analysis of PAHs.

### DNA extraction

DNA extraction from the glass fiber filter was performed following the method described in Yamamoto et al.^6^ using a DNeasy Blood and Tissue Kit (Qiagen, Hilde, Germany) and a Salivette tube (Sarstedt, Nümbrecht, Germany). Total eDNA was eluted in 100 μL AE buffer and preserved at − 20 °C.

DNA extraction from sediment was conducted using a combination of alkaline DNA extraction^45^ and ethanol precipitation, using a commercial soil DNA extraction kit (Power Soil DNA Isolation Kit, QIAGEN, Hilden, Germany), as described in Sakata et al.^26^. Wet sediment (ca. 3 g) was placed in a 15 mL tube. Triplicate samples were obtained from each petri dish in the tank experiment, and a single sample was obtained from each layer in the sediment cores. We added 6 mL of 0.33 M NaOH and 3 mL of 10 mM TE buffer (pH = 6.7) to the tube and mixed well using Voltex. The samples were incubated at 94 °C for 50 min, and during this time, they were inverted at 15 and 30 min of incubation. After the incubation, the samples were cooled for several minutes and then centrifuged at 5,000 × g for 30 s. Supernatants (7.5 mL) were collected in 50 mL tubes and 7.5 mL of 1 M Tris HCL buffer (pH = 9.0 in the tank experiment and pH = 6.7 in the core samples), 1.5 mL of 3 M sodium acetate (pH = 5.2), and 30 mL of 99.5% ethanol were added, and mixed well by inversion. Ethanol precipitation was achieved by incubating the mixture for 1 h at − 20 °C. As a negative control of extraction, 3 mL of pure water was treated in the same manner. Sediment in the tank experiment was processed up to the ethanol precipitation on the same day as sampling, whereas core samples were defrosted at room temperature prior to analysis and then preserved as precipitate.

The ethanol-precipitated sediment sample was centrifuged at 5,350 × g for 20 min, after which the supernatant was discarded. The precipitate was moved to the PowerBead Tube of the Power Soil Isolation Kit using a microspatula. The debris left in the centrifuged tube was also transferred by dissolving it in 100 μL of pure water. The following procedure was performed according to the protocol of Power Soil. The total eluted DNA (100 μL) was stored at − 20 °C. All the spatulas were bleached prior to use, and brand-new centrifugation tubes were used for the procedure.

### Quantitative PCR

DNA was quantified using real-time TaqMan PCR with a LightCycler 96 Real-Time PCR System (Roche, Basel, Switzerland). Species-specific sets of primers and probes were used to quantify the eDNA of jack mackerel, moon jellyfish, and sea nettle (Supplementary Table S5). For the specimens in the tank experiment, each reaction contained 2 μL of extracted eDNA solution, a final concentration of 900 nM of forward and reverse primers, and 125 nM of TaqMan probe in 1 × PCR master mix (FastStart Essential DNA Master; Roche, Basel, Switzerland). PCR was performed under the following conditions: 10 min at 95 °C, 50 cycles of 10 s at 95 °C, and 1 min at 60 °C. For the core samples, each reaction contained 5 μL of extracted eDNA solution, a final concentration of 900 nM of forward and reverse primers, and 125 nM of TaqMan probe in 1 × TaqMan Environmental Master Mix 2.0 (Thermo Fisher Scientific, Massachusetts, USA). PCR was performed under the following conditions: 2 min at 50 °C, 10 min at 95 °C, 60 cycles of 15 s at 95 °C, and 1 min at 60 °C. PCR was performed in triplicates for each extracted DNA sample. Triplicates of pure water instead of the eDNA solution were used for each PCR performance as a PCR negative control. All PCR negative controls were below the detection level.

As a standard for quantification, we used a linearized plasmid containing synthesized artificial DNA fragments of the cytochrome b (CytB) gene sequence of jack mackerel or cytochrome C oxidase subunit I (COI) gene sequences of moon jellyfish and Pacific sea nettle, including target regions. The dilution series of 3.0 × 10^1^–3.0 × 10^4^ was run in PCR in triplicate to obtain quantification curves. Quantification was accepted only when the fitted *R*^2^ value was above 0.99 on the quantification curve. The average of the PCR replicates was used to represent the eDNA concentration in each sample. eDNA concentrations were expressed as the number of copies per gram of samples in both water and sediment. As contamination precautions, water filtration, DNA extraction, and PCR reactions were performed in separate rooms, and persons entering one of the above three rooms were not permitted to enter the other rooms.

### Evaluation of PCR inhibitors

Sediment often contains chemicals that inhibit the PCR process. An analysis using an internal positive control (IPC) was conducted to confirm that the eDNA extraction kit successfully removed such inhibitive chemicals. DNA of lambda phage that was not present in the environment was used as the IPC^46^. Water and sediment samples (n = 12 for each) in the experimental tanks on day 14 after the introduction of fish were used for this experiment. We placed 300 copies of lambda phage DNA in the extracted eDNA with the primer–probe set in the test group, whereas pure water (instead of extracted eDNA) was placed in the control group (n = 3). PCR amplification of the test and control groups was compared, defining delta C_t_ as the difference in the number of threshold cycles (C_t_ values) in the PCR between samples with and without extracted eDNA. Delta C_t_ in the water samples ranged from − 0.49 to + 2.93 cycles, and from − 0.39 to + 0.28 cycles in the sediment samples (Supplementary Table S6). These values were less than + 3 cycles, previously proposed as criteria of inhibition^12^, and thus the inhibition was negligible in the present method.

### Analysis of polycyclic aromatic hydrocarbons (PAHs) for detecting tsunami signature

The sampled sediment cores (one at St. 1 and three at St. 3) were analyzed to quantify PAHs as a tsunami signature. Specimens from every two layers were used for the analysis.

Five hundred microliters of mixed acetone solution containing 5 μg mL^−1^ each of naphthalene-d_8_, acenaphthene-d_10_, fluorene-d_10_, anthracene-d_10_, fluoranthene-d_10_, pyrene-d_10_, and chrysene-d_12_ as surrogate standards was added to a centrifuge tube containing 1 g of sediment. The analytes were extracted twice by shaking for 10 min with acetone (10 mL). Supernatants mixed with 60 mL of saturated NaCl solution were transferred to a separatory funnel. The analytes were extracted twice with 10 mL hexane, and the organic layer was combined. This layer was then dried over anhydrous Na_2_SO_4_ and concentrated to trace level using a rotary evaporator. The solution was concentrated to 1 mL under a nitrogen atmosphere and cleaned using a Florisil Sep-Pak column (Waters Association Co., Ltd.). The Florisil Sep-Pak cartridge for clean-up was washed with 10 mL of hexane. A hexane solution containing the analytes followed by 10 mL of hexane/acetone (99/1) solution were passed through the prewashed cartridge. After the addition of 100 μL of 1 mg L^−1^ atradine-d5 as an internal standard, the eluate was carefully evaporated with a stream of nitrogen up to 1 mL. The analytes were determined using gas chromatography–mass spectrometry (GC/MS).

A Hewlett-Packard 6890 series gas chromatograph equipped with a mass spectrometer (5973 N) was used for PAH analysis. The separation was carried out in a capillary column coated with 5% phenyl methyl silicone (J&W Scientific Co., 30 m length × 0.25 mm i.d., 0.25 μm film thickness). The column temperature was maintained at 50 °C for the first minute and then increased to 290 °C at 20 °C min^−1^ and to 310 °C at 10 °C min^−1^. Finally, the column temperature was maintained at 310 °C for 10 min. The interface temperature, ion source temperature, and ion energy were 280 °C, 230 °C, and 70 eV, respectively. Selected ion monitoring was operated under this program. The monitoring ions of 128 (127) for naphthalene, 152 (151) for acenaphthylene, 153 (152) for acenaphthene, 166 (165) for fluorene, 178 (176) for phenanthrene and anthracene, 202 (203) for fluoranthene and pyrene, 228 (229) for benzo[a]anthracene and chrysene, 252 (253) for benzo[b]fluoranthene, 252 (281) for benzo[k]fluoranthene, and benzo[a]pyrene, 276 (207) for dibenzo[a,h]anthracene, indeno[1,2,3-cd]pyrene, and benzo[g,h,i]perylene, were used to quantify the concentrations of PAHs; qualifier ions are indicated in parentheses. One microliter of the sample was injected by splitless injection.

### Data analysis

Concentration of eDNA in the water and sediment of experimental tanks after the introduction of fish was analyzed by repeated-measures (rm) ANOVA; ‘days after the introduction of fish’ was defined as the explanatory variable, ‘concentration of eDNA’ as the response variable, and the ‘triplicates of petri dishes’ as a random factor. Then, eDNA concentrations among days were compared using Tukey’s HSD test. Homoscedasticity in eDNA content was improved by log _10_ (x + 1) transformation. The decrease in eDNA in water and sediment samples after the removal of fish was also analyzed by rm ANOVA in both the test and control tanks. A comparison of the eDNA concentrations between the test and control tanks was also conducted by rm ANOVA after the removal of fish. All analyses were performed in R ver. 3.4.2 (using the packages of lmerTest and multcomp)^47–49^.

Concentration of eDNA in water and sediment samples after introduction and removal of fish was fitted to eight candidate models as log X (y = a + b * ln(x)), log Y (y = exp(a + b * x)), asymptotic (y = a * x/(1 + b * x)), reciprocal (y = a + b/x), power law (y = a * x ^ b), exponential (y = a * exp(b * x)), and exponential decay (y = a + b * exp(c * x)) using the “nls” function of R. Models with the lowest AIC values were listed, and regression lines were drawn by Kaleida Graph 4.5 (Hulinks, Tokyo, Japan).

We tested whether the concentration of jellyfish eDNA was highest in the layers immediately above the signature of the tsunami in the sediment cores collected at St. 3. The depth of peak PAHs was identified in each core, and this was considered to represent the timing of the tsunami. Core samples of eDNA were then divided into the following three parts: (1) upper, including the upper half of the core above the PAH peak, representing recent sedimentation; (2) middle, including the lower half of the core above the PAH peak, representing sedimentation immediately after the tsunami; and (3) lower, including the layers of PAH peak and below, representing sedimentation at the timing of or prior to the tsunami. Concentrations of jellyfish eDNA of each species were compared among these three parts by nested ANOVA (layers nested in triplicate cores) followed by Tukey’s HSD test.

## Supplementary Information

Supplementary Information.

## Data Availability

All the data for the present study are presented in Supplementary tables.

## References

[1] Ficetola GF, Miaud C, Pompanon F, Taberlet P (2008). Species detection using environmental DNA from water samples. Biol. Lett..

[2] Takahara T, Minamoto T, Yamanaka H, Doi H, Kawabata Z (2012). Estimation of fish biomass using environmental DNA. PLoS ONE.

[3] Thomsen PF (2012). Monitoring endangered freshwater biodiversity using environmental DNA. Mol. Ecol..

[4] Miya M (2015). MiFish, a set of universal PCR primers for metabarcoding environmental DNA from fishes: Detection of more than 230 subtropical marine species. R. Soc. Open Sci..

[5] Deiner K, Fronhofer EA, Mächler E, Walser JC, Altermatt F (2016). Environmental DNA reveals that rivers are conveyer belts of biodiversity information. Nat. Commun..

[6] Yamamoto S (2017). Environmental DNA metabarcoding reveals local fish communities in a species-rich coastal sea. Sci. Rep..

[7] West KM (2020). Under the karst: Detecting hidden subterranean assemblages using eDNA metabarcoding in the caves of Christmas Island, Australia. Sci. Rep..

[8] Barnes MA (2014). Environmental conditions influence eDNA persistence in aquatic systems. Environ. Sci. Technol..

[9] Maruyama A, Nakamura K, Yamanaka H, Kondoh M, Minamoto T (2014). The release rate of environmental DNA from juvenile and adult fish. PLoS ONE.

[10] Thomsen PF (2012). Detection of a diverse marine fish fauna using environmental DNA from seawater samples. PLoS ONE.

[11] Murakami H (2019). Dispersion and degradation of environmental DNA from caged fish in a marine environment. Fish. Sci..

[12] Turner CR, Uy KL, Everhart RC (2015). Fish environmental DNA is more concentrated in aquatic sediments than surface water. Biol. Conserv..

[13] Anderson-Carpenter LL (2011). Ancient DNA from lake sediments: Bridging the gap between paleoecology and genetics. BMC Evol. Biol..

[14] Pedersen MW (2013). A comparative study of ancient environmental DNA to pollen and macrofossils from lake sediments reveals taxonomic overlap and additional plant taxa. Quat. Sci. Rev..

[15] Shaw JLA (2016). Comparison of environmental DNA metabarcoding and conventional fish survey methods in a river system. Biol. Conserv..

[16] Sakata MK (2020). Determining an effective sampling method for eDNA metabarcoding: a case study for fish biodiversity monitoring in a small, natural river. Limnology.

[17] McDevitt AD (2019). Environmental DNA metabarcoding as an effective and rapid tool for fish monitoring in canals. J. Fish Biol..

[18] Laroche O, Kersten O, Smith CR, Goetze E (2020). Environmental DNA surveys detect distinct metazoan communities across abyssal plains and seamounts in the western Clarion Clipperton Zone. Mol. Ecol..

[19] Brandt M (2021). Evaluating sediment and water sampling methods for the estimation of deep-sea biodiversity using environmental DNA. Sci. Rep..

[20] Govindarajan AF (2021). Exploring the use of environmental DNA (eDNA) to detect animal taxa in the mesopelagic zone. Front. Ecol. Evol..

[21] Wei N, Nakajima F, Tobino T (2019). Variation of environmental DNA in sediment at different temporal scales in nearshore area of Tokyo Bay. J. Water Environ. Technol..

[22] Kuwae M (2020). Sedimentary DNA tracks decadal-centennial changes in fish abundance. Commun. Biol..

[23] Kozil A (2019). Environmental DNA metabarcoding studies are critically affected by substrate selection. Mol. Ecol. Res..

[24] Holman LE (2019). Detection of introduced and resident marine species using environmental DNA metabarcoding of sediment and water. Sci. Rep..

[25] Van den Heuvel-Greve MJ (2021). Early detection of marine non-indigenous species on Svalbard by DNA metabarcoding of sediment. Polar Biol..

[26] Sakata MK (2020). Sedimentary eDNA provides different information on timescale and fish species composition compared with aqueous eDNA. Environ. DNA.

[27] Wei N, Nakajima F, Tobino T (2018). A microcosm study of surface environmental DNA: Decay observation, abundance estimation, and fragment length comparison. Environ. Sci. Technol..

[28] Nishino T, Imazu Y (2018). A computational model for large-scale oil spill fires on water in tsunamis: Simulation of oil fires at Kesennuma Bay in the 2011 Great East Japan Earthquake and Tsunami. J. Loss Prevent. Proc..

[29] Masuda R, Hatakeyama M, Yokoyama K, Tanaka M (2016). Recovery of coastal fauna after the 2011 tsunami in Japan as determined by bimonthly underwater visual censuses conducted over five years. PLoS ONE.

[30] Takahashi S, Sakata MK, Minamoto T, Masuda R (2020). Comparing the efficiency of open and enclosed filtration systems in environmental DNA quantification for fish and jellyfish. PLoS ONE.

[31] Kahkashan S (2019). Evaluation of marine sediment contamination by polycyclic aromatic hydrocarbons along the Karachi coast, Pakistan, 11 years after the Tasman Spirit oil spill. Chemosphere.

[32] Pedersen MW (2015). Ancient and modern environmental DNA. Philos. Trans. R. Soc. B..

[33] Horiuchi T, Masuda R, Murakami H, Yamamoto S, Minamoto T (2019). Biomass-dependent emission of environmental DNA in jack mackerel *Trachurus japonicus* juveniles. J. Fish Biol..

[34] Corinaldesi C, Beolchini F, Dell'Anno A (2008). Damage and degradation rates of extracellular DNA in marine sediments: Implications for the preservation of gene sequences. Mol. Ecol..

[35] Corinaldesi C, Barucca M, Luna GM, Dell'Anno A (2011). Preservation, origin and genetic imprint of extracellular DNA in permanently anoxic deep-sea sediments. Mol. Ecol..

[36] Tori A, Lever MA, Jørgensen BB (2015). Origin, dynamics, and implications of extracellular DNA pools in marine sediments. Mar. Genom..

[37] Minamoto T (2017). Environmental DNA reflects spatial and temporal jellyfish distribution. PLoS ONE.

[38] Masuda R (2012). Underwater visual census of fish assemblages in Moune Bay, Kesennuma. Aquabiology.

[39] Suzuki KS (2019). Seasonal alternation of the ontogenetic development of the moon jellyfish *Aurelia coerulea* in Maizuru Bay, Japan. PLoS ONE.

[40] Shoji J, Masuda R, Yamashita Y, Tanaka M (2005). Effect of low dissolved oxygen concentrations on behavior and predation rates on red sea bream *Pagrus major* larvae by the jellyfish *Aurelia aurita* and by juvenile Spanish mackerel *Scomberomorus niphonius*. Mar. Biol..

[41] Pitt KA, Kingsford MJ, Rissik D, Koop K (2007). Jellyfish modify the response of planktonic assemblages to nutrient pulses. Mar. Ecol. Prog. Ser..

[42] Ueda K, Torigoe H (2012). Why do victims of the tsunami return to the coast?. Int. J. Japan. Sociobiol..

[43] Masuda R (2008). Seasonal and interannual variation of subtidal fish assemblages in Wakasa Bay with reference to the warming trend in the Sea of Japan. Environ. Biol. Fish..

[44] Tsuji S, Takahara T, Doi H, Shibata N, Yamanaka H (2019). The detection of aquatic macroorganisms using environmental DNA analysis—A review of methods for collection, extraction, and detection. Environ. DNA.

[45] Kouduka M (2012). A new DNA extraction method by controlled alkaline treatments from consolidated subsurface sediments. FEMS Microbiol. Lett..

[46] Minamoto T (2009). Detection of cyprinid herpesvirus 3 DNA in river water during and after an outbreak. Vet. Microbiol..

[47] R Core Team (2017). R: A Language and Environment for Statistical Computing.

[48] Kuznetsova A, Brockhoff PB, Christensen RHB (2017). lmerTest package: Tests in linear mixed effects models. J. Stat. Softw..

[49] Hothorn T, Bretz F, Westfall P (2008). Simultaneous inference in general parametric models. Biom. J..

